# Impact of a Polymer-Based Nanoparticle with Formoterol Drug as Nanocarrier System In Vitro and in an Experimental Asthmatic Model

**DOI:** 10.3390/toxics11120974

**Published:** 2023-11-30

**Authors:** Buket Bakan, Anne-Charlotte Jonckheere, Tatjana Decaesteker, Nora F. Marain, Sivakumar Murugadoss, Nefise Ulku Karabay Yavasoglu, Umut Şahar, Raziye Hilal Şenay, Sinan Akgöl, Özlem Göksel, Peter H. M. Hoet, Jeroen A. J. Vanoirbeek

**Affiliations:** 1 Department of Public Health and Primary Care, Centre for Environment and Health, KU Leuven, 3000 Leuven, Belgium; buketbakan@gmail.com (B.B.); peter.hoet@kuleuven.be (P.H.M.H.); 2 Department of Molecular Biology and Genetics, Faculty of Science, Atatürk University, Erzurum 25240, Turkey; 3 Allergy and Clinical Immunology Research Group, Department of Microbiology, Immunology and Transplantation, KU Leuven, 3000 Leuven, Belgium; 4 BREATH, Department of Chronic Diseases and Metabolism, KU Leuven, 3000 Leuven, Belgiumnora.marain@kuleuven.be (N.F.M.); 5 Department of Biology, Faculty of Science, Ege University, Izmir 35100, Turkey; ulku.karabay@ege.edu.tr (N.U.K.Y.); umut.sahar@ege.edu.tr (U.Ş.); 6 Department of Biochemistry, Faculty of Science, Ege University, Izmir 35100, Turkey; hilalsenay@gmail.com (R.H.Ş.); sinan.akgol@ege.edu.tr (S.A.); 7 Laboratory of Occupational & Environmental Respiratory Diseases and Asthma, Ege University, Izmir 35040, Turkey; goksel.ozlem@gmail.com

**Keywords:** nano drug carrier, in vitro, formoterol, asthma, mice

## Abstract

The implementation of nanotechnology in pulmonary delivery systems might result in better and more specific therapy. Therefore, a nano-sized drug carrier should be toxicologically inert and not induce adverse effects. We aimed to investigate the responses of a polymer nano drug carrier, a lysine poly-hydroxyethyl methacrylate nanoparticle (NP) [Lys-p(HEMA)], loaded with formoterol, both in vitro and in vivo in an ovalbumin (OVA) asthma model. The successfully synthesized nanodrug formulation showed an expectedly steady in vitro release profile. There was no sign of in vitro toxicity, and the 16HBE and THP-1 cell lines remained vital after exposure to the nanocarrier, both loaded and unloaded. In an experimental asthma model (Balb/c mice) of ovalbumin sensitization and challenge, the nanocarrier loaded and unloaded with formoterol was tested in a preventive strategy and compared to treatment with the drug in a normal formulation. The airway hyperresponsiveness (AHR) and pulmonary inflammation in the bronchoalveolar lavage (BAL), both cellular and biochemical, were assessed. The application of formoterol as a regular drug and the unloaded and formoterol-loaded NP in OVA-sensitized mice followed by a saline challenge was not different from the control group. Yet, both the NP formulation and the normal drug application led to a more deteriorated lung function and increased lung inflammation in the OVA-sensitized and -challenged mice, showing that the use of the p(HEMA) nanocarrier loaded with formoterol needs more extensive testing before it can be applied in clinical settings.

## 1. Introduction

Asthma is a chronic airway disease characterized by reversible airway obstruction, chronic inflammation, and non-specific bronchial hyperreactivity. Asthma is a worldwide health problem with an increasing prevalence in many countries, affecting around 300 million individuals [1,2,3]. Many immune cells are involved in the pathogenesis of asthma, including neutrophils, macrophages, eosinophils, mast cells, dendritic cells, and T and B lymphocytes [4]. In asthma, two distinct endotypes (Th2 and non-Th2) are commonly described. Both of them can be further differentiated into phenotypes (Th2: atopic, late onset, aspirin-exacerbated respiratory disease; non-Th2: non-atopic, obesity related, elderly related, exercise-induced asthma, etc.) [5]. Depending on the phenotype of asthma, long-acting β-agonists (LABAs) and short-acting β-agonists (SABAs) are the most common treatment in combination with an inhaled corticosteroid (ICS) [6,7]. However, high doses of ICS may induce the risk of side effects in the long term [8,9]. Formoterol is a potent and selective long-acting β2-agonist, with a significantly faster initiation of the bronchodilation effect [10]. Treatments with this type of drug aim to mediate inflammation and ease obstructive symptoms, yet the effect on chronic airway remodeling is very limited.

Recent advances in the field of nanotechnology are aiming to improve the diagnosis and treatment of diseases [11]. There are already nanodrug formulations commercially approved by the US FDA [12]. One of the applications for which nanoparticles are used is drug delivery. Nanoparticles as a drug delivery system have multiple advantages, such as improved bioavailability, the carrying of insoluble drugs, reduced side effects, targeted therapy, and increased permeability to biological membranes in different routes of administration [13,14]. Especially, polymer-based nanoparticles are used as drug delivery systems due to the prolonged duration of their presence in the lungs [15]. There are also downsides to nanoparticle use for drug delivery systems, concerning systemic uptake, mucosal clearance, being a causal factor of inflammation due to the design of the nano-sized aerosol particles in pulmonary delivery, among others [16]. Yet, these limitations do not outweigh the advantages, resulting in the rapidly increasing use of nanoparticles in the diagnosis and treatment of many diseases [17,18].

Poly-hydroxyethyl methacrylate p(HEMA) as a synthetic polymer has the potential to be used as a drug delivery system because of its high purity, ease of tailoring and good chemical stability over the natural polymers [19]. Lys-p(HEMA) has hydroxyl and amine groups at the surface of its nanoparticle. Specifically, the amine groups allow for the binding of other functional groups that exist in drugs. Previously, Bakan et al. (2019) already showed the biocompatibility of Lys-p(HEMA) due to these physico-chemical properties [20].

We aimed to demonstrate the effectiveness of nanoparticles based on the polymer nanomaterial Lys-p(HEMA) as a drug carrier, loaded with formoterol, since, in the field of asthma, only a limited number of studies on drug delivery with nano-based materials are available. To investigate our aim, we exposed in vitro 16-HBE and THP-1 cell lines to the nanoparticle carrier to determine its cytotoxicity. Subsequently, we investigated the potential drug efficacy of the nanocarrier loaded with formoterol on airway inflammation and airway hyperreactivity in a mouse model of ovalbumin-induced asthma.

## 2. Material and Methods

### 2.1. Reagents

2-hydroxyethyl methacrylate (HEMA), ethylene glycol di-methacrylate (EGDMA) and the amino acid lysine were purchased from Sigma Chem. Co. (St. Louis, MO, USA). Ovalbumin was obtained from Sigma-Aldrich (St. Louis, MO, USA, Cat No 9006-59-1) and alum adjuvant from ThermoFisher Company, (Waltham, MA, USA, catalog number: 77161). Pentobarbital (Nembutal) was obtained from Sanofi Sante’ Animale (CEVA, Brussels, Belgium) and Isoflurane (Forene1) from Abbott Laboratories (SA Abbott NV, Ottignies, Belgium). All other chemicals were obtained from Sigma-Aldrich.

### 2.2. Synthesis and Characterization Analyses

#### 2.2.1. Synthesis of Lys-p(HEMA) Nanoparticles

Lys-p(HEMA) nanoparticles (NP) were synthesized with surfactant-free emulsion polymerization and grafting methods according to the protocol in Bakan et al. (2019) [20]. After the polymerization step, in order to prepare lysine-grafted poly(HEMA) polymeric NPs, firstly, 20 g of dry poly(HEMA) was weighed and transferred into the reactor. Lysine solution was prepared with 3 g lysine in 50 mL of tetrahydrofuran and 1.4 g of NaH as a catalyst was added to the solution. Constant gentle magnetic stirring was used for the grafting reaction at 40 °C for 24 h. After the reaction period, lysine-grafted polymer was removed and washed with water and methanol to remove unreacted molecules and dried in vacuum for 24 h. The samples were stored at 4 °C until further use [20].

Characterization analyses were performed by using Zeta-Sizer, scanning electron microscopy (SEM) analysis, and Fourier-transform infrared spectroscopy (FT-IR) and the results were presented in our previous study. Lys-p(HEMA) NPs has an average size of around 171 nm, the zeta potential was −22.6 mV, and the characteristic peaks of the stretching band were observed in the FT-IR spectrum [20].

#### 2.2.2. Synthesis of Lys-p(HEMA) NPs–Formoterol Formulation

Drug loading onto Lys-p(HEMA) NPs was carried out with 250 µg/mL formoterol concentration in ethanol at room temperature for 1 h. Lys-p(HEMA) NPs were centrifuged at 30,000× *g* and formoterol solution was removed from the NPs. The amount of binding of formoterol onto Lys-p(HEMA) NPs was calculated using Equation (1).

(1) $$Q=\frac{\left(Ci-Cf\right)x\;V}{m}$$

*Q* is the amount of bound formoterol (µg) per mg mass of nanopolymer (µg/mg); initial and final formoterol concentrations were demonstrated as *Ci* and *Cf* (µg/mL); *V* is the total volume of the solution (mL); and *m* is the mass of the nanopolymer used in the experiment (mg).

### 2.3. Cell Culture and Cytotoxicity Assay of Lys-p(HEMA) NPs

The human bronchial epithelial cell line (16HBE) and human monocytic cell line (THP-1) were provided by Dr. Gruenert (University of California, San Francisco, CA, USA). THP-1 cells were cultured in RPMI1640 supplemented with 10% fetal bovine serum (FBS), 100 U/mL penicillin/streptomycin, 2 mM L-glutamine, and 2.5 μg/mL fungizone. 16HBE cells were cultured in DMEM/F12 supplemented with 5% FBS, 100 U/mL penicillin/streptomycin, 2 mM L-glutamine, and 2.5 μg/mL fungizone. Cells were incubated at 37 °C in 100% humidified air containing 5% CO_2_. The culture was changed every 2 or 3 days until cells were confluent. Cytotoxicity tests were performed with a standard protocol [21,22]. The cultured cells were treated with various concentrations of NPs (6.25 μg to 1 mg/mL) and incubated for 24 h at 37 °C. In a WST-1 assay, the optical density was measured at 450 nm in a multi-well plate reader (BIORAD, Model680XR microplate reader). The relative viability was calculated against the negative controls (untreated cells). Potential morphology changes in cells treated with different NP concentrations were examined using inverted light microscopy.

The percentage cell viability was determined using Equation (2) [23]:

(2) $$viable\;cells\%=\frac{\left(absorbance\;of\;treated\;cells\right)-(absorbance\;of\;the\;blank)}{\left(absorbance\;of\;the\;control\right)-(absorbance\;of\;the\;blank)}\times 100$$

On the LDH assay, the change in absorbance (depletion of NADH) was measured using spectrophotometry at 340 nm for 3 min with 15 sec intervals. Cell viability was determined using Equations (3) and (4).

(3) $$cell\;viability\;\%=\frac{\left(slope\;of\;lysate\right)}{\left(slope\;of\;lysate+slope\;of\;supernatant\right)}\times 100$$

(4) $$relative\;cell\;viability\;\%=\frac{\left(sample\;viability\right)}{\left(untreated\;control\;viability\right)}\times 100$$

### 2.4. In Vitro Release of Formoterol from the Nanocarrier

Using an Agilent 1200 Capillary HPLC and Bruker HCT Ultra Ion Trap MS^n^ system with simulated lung fluids (SLFs) release medium, we have determined the in vitro release capacity of formoterol from the nanoparticle Lys-p(HEMA) carrier. We made a 5-point calibration solution of formoterol (100, 250, 500, 750, and 1000 ng/mL) bound to Lys-p(HEMA) NPs. Next, the Lys-p(HEMA) NPs were transferred into cellulose dialysis membrane and formoterol release was started in SLF at 37 °C. Formoterol release was measured using Agilent 1200 Capillary HPLC and Bruker HCT Ultra Ion Trap MSn system according to the time interval. Analytic separation of formoterol loaded on the Lys-p(HEMA) NPs was performed using the Agilent 1200 Capillary HPLC, with a narrow-bore C8 column (ACE 150 × 0.5 mm 5 μm) at room temperature on isocratic elution mode. Formic acid (mobile phase A, 0.1% (*v*/*v*)) and acetonitrile (mobile phase B) were used as a mobile phase. The elution was run using an isocratic elution of 1:1 (*v*/*v*) of A and B for 5 min. Sample injection volume and flow rate were adjusted to 0.5 μL and 20 μL/min, respectively. Mass spectrometry detection was performed using Bruker HCT Ultra ion trap MS (Bruker Daltonics, Bremen, Germany) in the positive mode, with an *m*/*z* range of 100–500. Formoterol-loaded Lys-p(HEMA) NPs were analyzed in the positive mode, and the precursor ions had an *m*/*z* of 345.2. The most intense fragment ion at *m*/*z* 327.2 was used for quantification. The other fragment ions were observed at *m*/*z* 120.9 and 148.9. In order to understand the stability and shelf-life of formoterol-loaded Lys-p(HEMA) NPs, they were stored for 10 days at +4 °C.

### 2.5. Asthma Mouse Model

The experimental procedures performed on mice were approved by the KU Leuven Local Ethical Committee for animal experiments (n°. 094/2018). Male BALB/c mice (6–7 weeks/20–25 g) were housed in an optimal air-conditioned room in a 12 h light–dark cycle. Food and water were available ad libitum.

#### 2.5.1. Experimental Protocol of OVA-Induced Asthma Model

**Figure 1:**
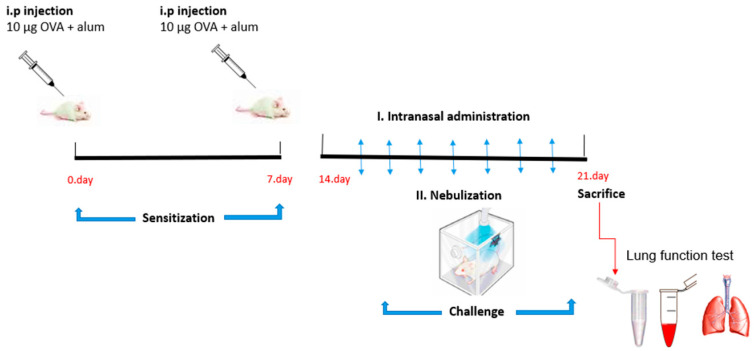
The experimental design in BALB/c mice with time points of sensitization, challenge, administration of test samples, and outcomes.

Eight groups of mice were designated as the experimental groups (*n* = 6) and animals were divided into groups randomly (OVA/sal-sal; OVA/NP-sal; OVA/sal-OVA; OVA/NP-OVA; OVA/ND-sal; OVA/ND-OVA; OVA/D-sal; OVA/D-OVA). Figure 1 shows the treatment scheme of the mice. On days 0 and 7, all mice were sensitized by intraperitoneal injection of 10 µg OVA+ 1 mg alum. From day 14 to day 20, the mice received, on a daily basis, an intranasal instillation (20 µg in 50 µL), under isoflurane anesthesia, with 0.9% NaCl saline (sal), unloaded nanoparticles (NP), formoterol drug in solution (D), or the nanocarrier loaded with formoterol (nanodrug—ND). Each day, 10 min after the intranasal administration, the mice received an aerosol challenge with 1% OVA or saline for 20 min. On day 21, lung function assessments were performed, followed by an autopsy.

#### 2.5.2. Lung Function Measurement

Lung function measurements were assessed 24 h after the last intranasal instillation and aerosol challenge, using a forced oscillation technique (FlexiVent; SCIREQ, Montreal, QC, Canada) [24]. Mice were deeply anesthetized by an intraperitoneal injection of pentobarbital sodium (70 mg/kg body weight) (Nembutal, Abbott Laboratories, Madrid, Spain), the trachea was exposed, tracheotomized, and connected to the ventilator. First baseline measurements were performed, using the quick-prime 3 perturbation (QP3) to determine the airway resistance (Rn), tissue elasticity (H), and the tissue damping (G), along with the negative pressure forced expiration (NPFE) perturbation, to determine the forced vital capacity (FVC) and the forced expiratory volume in 0.1 s (FEV_0.1_). Each plotted value is the average of 3 measurements. Following the baseline measurements, the mice received increasing concentrations of methacholine (0 to 20 mg/mL) to determine the airway hyperreactivity (AHR). Both the QP3 and NPFE perturbation was used to assess the Rn (QP3) and the FEV_0.1_ (NPFE). For the AHR plotted as Rn, the area under the curve of the dose response is calculated, while for the AHR plotted as FEV_0.1_, the PC_20_ (provocative concentration leading to a 20% decrease in FEV_0.1_) is calculated.

#### 2.5.3. Lung Inflammation

After the lung function measurements, the mice received an overdose of pentobarbital and an autopsy was performed. The lungs were lavaged three times with 0.7 mL sterile saline (0.9% NaCl), and the bronchoalveolar lavage (BAL) fluid was collected. The total cell number was counted using a Bürker hemocytometer and the bronchoalveolar lavage (BAL) fluid was centrifuged at 1000× *g*, for 10 min. For differential cell counts, 250 μL of the resuspended cells (100,000 cells/mL) were spun (1400× *g*, 6 min) (Cytospin 3, Shandon, TechGen, Zellik, Belgium) and applied to microscope slides, air-dried, and stained using the Diff-Quik^®^ method (Medical Diagnostics, Düdingen, Germany). In the experiment, 200 cells were counted for the number of macrophages, eosinophils, neutrophils, and lymphocytes in each sample.

In the BAL fluid, we assessed the concentration of the following cytokines and chemokines: interferon-g (IFN-g), IL-13, IL-17A, IL-4, IL-5, IL-6, IL-33, IL-17F, keratinocyte-derived chemokine (KC), and tumor necrosis factor-a (TNF-a), using a U-plex Assay (Meso Scale Diagnostics, Rockville, Maryland, USA) according to the manufacturer’s instruction. The detection limits were 0.206 pg/mL, 6.26 pg/mL, 0.0785 pg/mL, 0.289 pg/mL, 0.225 pg/mL, 3.53 pg/mL, 0.184 pg/mL, 32.6 pg/mL, 0.152 pg/mL, and 0.280 pg/mL, respectively.

### 2.6. Data Analysis

The experimental data is expressed as mean ± standard deviation (SD) or as mouse individual data with the group mean. One-way analysis of variance (ANOVA) followed by Tukey’s test was performed for comparison between the groups (GraphPad Prism 8.02). *p* < 0.05 was considered as the statistical level of significance.

## 3. Results

### 3.1. Synthesis of Lys-p(HEMA) NPs–Formoterol Formulation

For the design of the nano-based drug treatment, the Lys-p(HEMA) NPs were synthesized by the surfactant-free polymerization technique. The carboxyl group of p(HEMA) was bound to an amino group of Lys and the graft yield of the Lys-p(HEMA) NPs was calculated as 59%. Then, the NPs were loaded with formoterol, which is commonly used in the treatment of asthma.

Drug loading onto Lys-p(HEMA) NPs–formoterol was carried out successfully and the amount of formoterol bound onto the Lys-p(HEMA) NPs’ value (Q) was calculated as 180.62 µg/mg.

### 3.2. Cytotoxicity Test Results

**Figure 2:**
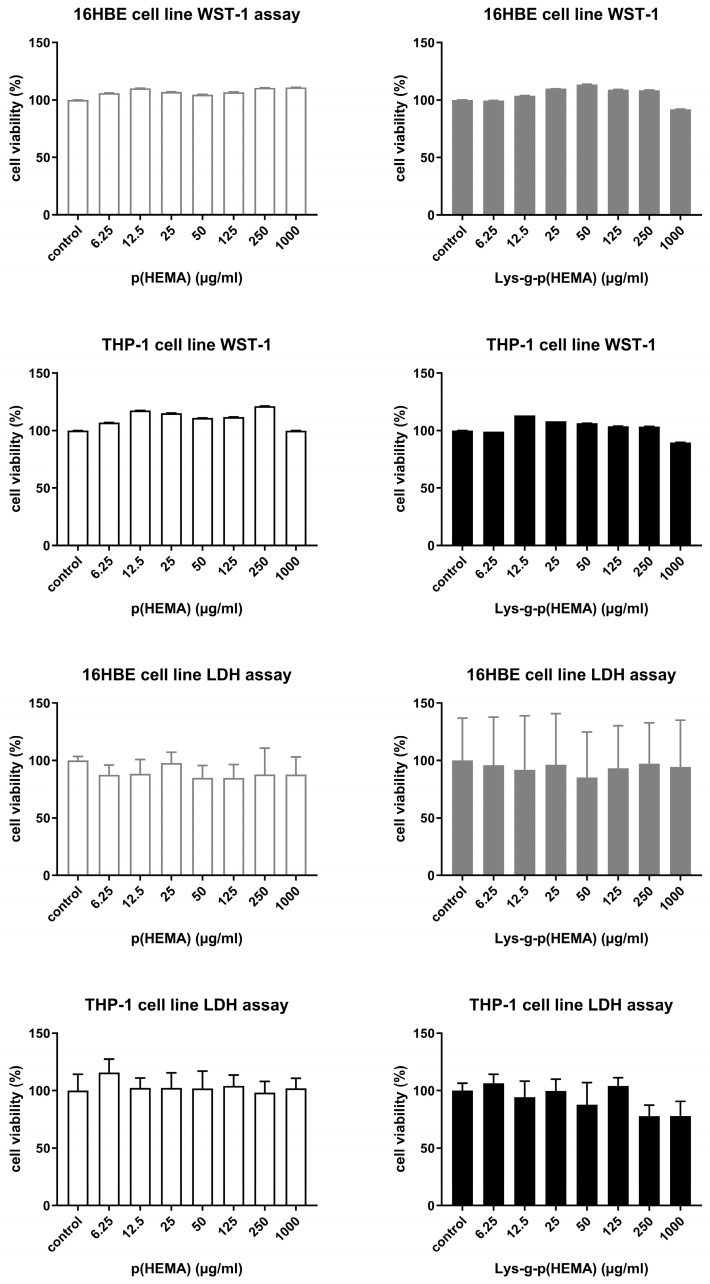
Cytotoxicity testing: WST-1 and LDH assay after exposure to p(HEMA) and Lys-p(HEMA) NPs at different concentrations to 16HBE and THP-1 cells. Data are mean ± SD from three repeats and three independent experiments.

The Lys-p(HEMA) NPs did not show cytotoxic effects at any concentrations after 24 h incubation in the 16HBE and THP-1 cell lines in the WST-1 and LDH assays (Figure 2). The cells were homogeneously distributed on the culture flasks, and in both the control and exposure groups, no morphological change was obtained on both cell lines after the NP treatment.

### 3.3. In Vitro Release Profile Results

For the method’s performance and validation, a five-point calibration curve was constructed. The regression coefficient for this calibration curve was higher than 0.99. The three replicates of a ‘low’ formoterol concentration (100 ng/mL) were analyzed and the limit of quantitation (LOQ) was calculated to be 41.9 ng/mL, according to the signal/noise ratio. The relative standard deviation for the three repetitions of this sample was 3.4 ng/mL. The precision, presented as the percentage of the relative standard deviation (RSD, %) was 3.31% for 500 ng/mL of formoterol. The accuracy (recovery studies) was calculated using the initial and final concentration ratio, and the mean recovery was 100.8%.

**Figure 3:**
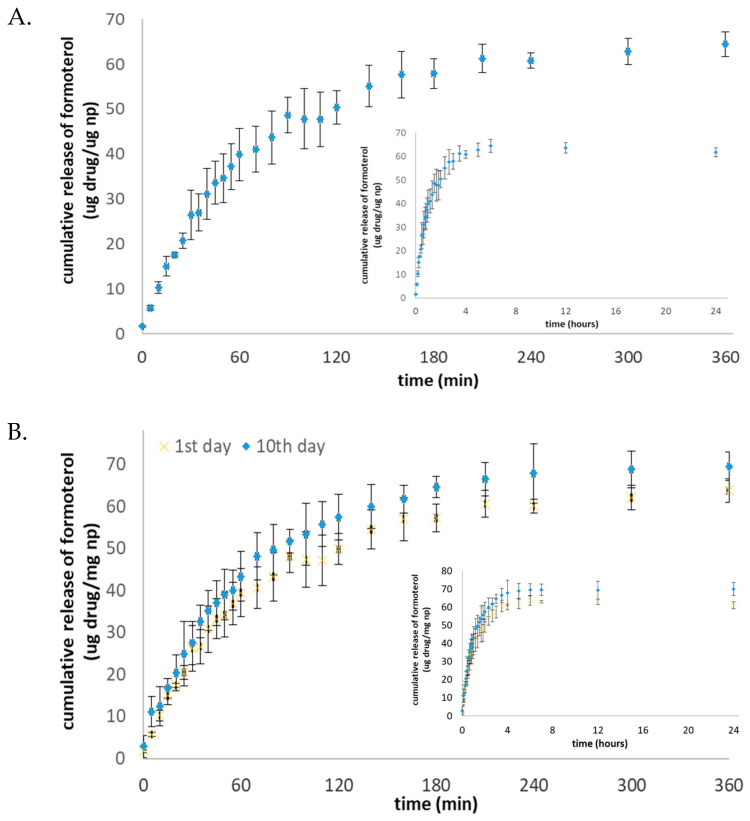
(**A**) In vitro release profile of Lys-p(HEMA) NPs–formoterol formulation in SLF; (**B**) in vitro release profile of Lys-p(HEMA) NPs–formoterol formulation after being stored for 10 days at +4 °C. Large figures are the release profiles over 360 min; the insert figures are the release profiles over 24 h. Data are mean ± SD (*n* = 3).

Figure 3 shows the in vitro release pattern of the Lys-p(HEMA) NPs–formoterol formulation in the simulated lung fluids (SLFs). Initially, there was a burst release of the drug, which lasted up to 120 min, after which the drug release was more stable and sustainable in the SLF. Upon the storage of Lys-p(HEMA) NPs–formoterol formulation at 4 °C for 10 days, the drug release profile was like that of freshly prepared solution.

### 3.4. OVA-Induced Asthma Model

#### 3.4.1. Organ Weights of Mice

**Figure 4:**
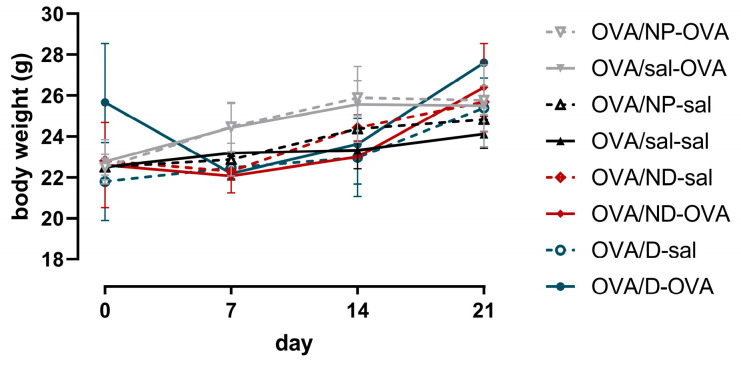
Body weights of mice in all groups. One-way ANOVA was used to compare the control and treatment groups. OVA: ovalbumin; sal: saline; NP: nanoparticle; ND: nanodrug formulation (Lys-p(HEMA) NP–formoterol); D: drug. Data are presented as mean ± SD (*n* = 6).

Over the course of the experiment, the body weights of the mice did not statistically differ between the treatment and control groups (Figure 4).

#### 3.4.2. Lung Function Measurement Results

**Figure 5:**
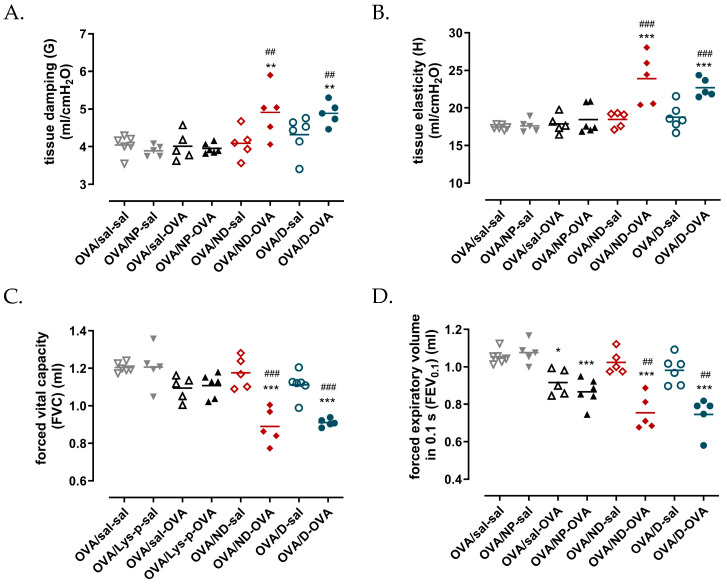
Baseline lung function. Tissue damping (**A**) and tissue elasticity (**B**) were measured using the forced oscillation perturbation QP3, while the forced vital capacity (**C**) and forced expiratory volume in 0.1 s (**D**) were assessed using the NPFE perturbation. OVA: ovalbumin; sal: saline; NP: nanoparticle; ND: nanodrug formulation (Lys-p(HEMA) NP–formoterol); D: drug. Data are presented as the individual result and the group mean (*n* = 5–6), * *p* < 0.05, ** *p* < 0.01, *** *p* < 0.001 compared to OVA/sal/sal and ^##^ *p* < 0.01, ^###^ *p* < 0.001 compared to OVA/sal-OVA.

One day after the last ovalbumin aerosol challenge, the mice were anesthetized, and the lung function was assessed. Figure 5 shows the baseline lung function parameters measured using the QP3 and NPFE perturbation. At baseline, ovalbumin sensitization and challenge with both the drug in the nanocarrier (OVA/ND-OVA) and as a drug in a normal formulation (OVA/D-OVA) resulted in a significantly increased tissue damping (G) (Figure 5A) and tissue elasticity (H) (Figure 5B), while the airway resistance did not differ compared to the OVA/sal-sal control groups and OVA/sal-OVA positive control groups. The FVC and the FEV_0.1_ of the OVA/ND-OVA and OVA/D-OVA groups were statistically lower compared to the negative control group OVA/sal-sal and the OVA/sal-OVA positive control group (Figure 5C,D), while, for the FEV_0.1_, the other two OVA-challenged groups (OVA/sal/OVA and OVA/NP-OVA) were statistically lower compared to the OVA/sal-sal control group (Figure 5D).

**Figure 6:**
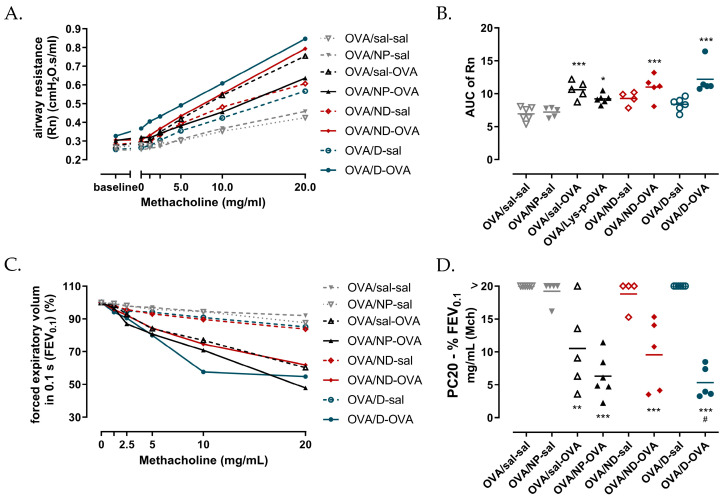
Airway hyperreactivity to methacholine challenge. Airway resistance (**A**) is measured using the QP3 perturbation; afterwards, the area under the curve (AUC) of each individual mouse is calculated (**B**). FEV_0.1_ (**C**) is measured using the NPFE perturbation directly after the last QP3 measurement. PC20 is the concentration at which the FEV_0.1_ decreased 20% from the baseline (**D**). OVA: ovalbumin; sal: saline; NP: nanoparticle; ND: nanodrug formulation (Lys-p(HEMA) NP–formoterol); D: drug. The dose–response data (**A**,**C**) are presented as group means, while the AUC of the Rn and the PC20 data (**B**,**D**) are presented as the individual result and the group mean (*n* = 5–6). * *p* < 0.05, ** *p* < 0.01, *** *p* < 0.001 compared to OVA/sal/sal and ^#^ *p* < 0.05 compared to OVA/sal-OVA.

After the baseline measurements, we initiated a methacholine provocation protocol to assess the airway hyperreactivity (Figure 6). Upon methacholine provocation, the airway resistance (Rn) of all the OVA-challenged groups increased significantly compared to the OVA/sal-sal control group, while there was no difference in the AHR between the positive control OVA/sal-OVA and the OVA/ND-OVA and OVA/D-OVA groups (Figure 6A,B). Similar results were found with the NPFE perturbation, as shown by the FEV_0.1_ response to methacholine and subsequent calculation of the PC_20_ (Figure 6C,D).

#### 3.4.3. Bronchoalveolar Lavage Fluid Results

**Figure 7:**
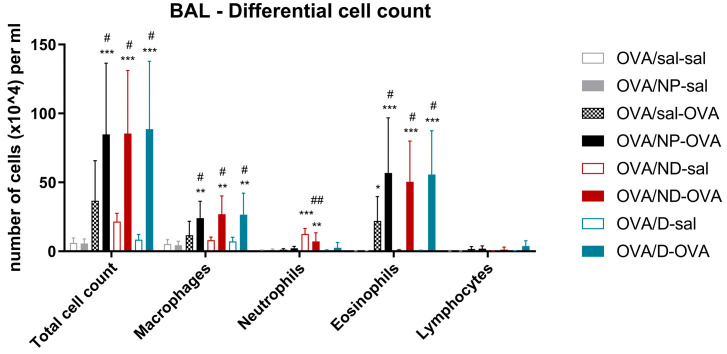
Total and differential bronco-alveolar lavage (BAL) cell counts. BAL fluid was collected 24 h after the last intranasal administration and OVA challenge. OVA: ovalbumin; sal: saline; NP: nanoparticle; ND: nanodrug formulation (Lys-p(HEMA) NP–formoterol); D: drug. Data are presented as mean ± SD (*n* = 5–6). * *p* < 0.05, ** *p* < 0.01, *** *p* < 0.001 compared to OVA/sal/sal and ^#^ *p* < 0.05, ^##^ *p* < 0.01 compared to OVA/sal-OVA.

Figure 7 shows the total and differential cell counts of the BAL. The OVA-challenged groups had a significantly higher number of cells in the BAL compared with the OVA/sal-sal negative control group. The OVA-sensitized and -challenged groups that received the NP, ND, or the D had a statistically higher number of macrophages and eosinophils compared to the positive OVA/sal-OVA group. The two groups that received the ND (OVA/ND-sal and OVA/ND-OVA showed a significantly higher number of neutrophils in their BAL, compared to the OVA/sal-sal negative control group, while the OVA-ND-sal had a statistically higher number of neutrophils compared to the positive OVA/sal-OVA group.

Table 1 shows the results of the cytokine analysis in the BAL fluid. Since the concentrations of several cytokines in the control group OVA/sal-sal are often below the LOD, not all the statistical analyses could be performed. All the Th2-related cytokines (IL-4, IL-5, and IL-13) and IL17A were abundantly present in the all OVA-sensitized and -challenged groups. IL-4 was below the LOD in all the saline-challenged groups. This was not the case for IL-5 (present in OVA/ND-sal and OVA/D-sal), IL 13 (present in OVA/D/sal), and IL-17A (present in OVA/ND-sal). In contrary to IL17A, IL17F was only detected in two saline challenge groups (OVA/ND-sal and OVA-D-sal). For IL33, there was only a significant difference between the positive control OVA/sal-OVA and the OVA-sensitized and OVA-challenged group that received NPs without formoterol (OVA/NP-OVA). The Th1 cytokine, IFN-γ, and the systemic inflammation marker, IL-6, were only detectable in the OVA/ND-OVA group. TNF-α was significantly increased in the OVA/ND-sal group, compared to the OVA/sal-sal control group. Almost all groups (except the OVA/D-sal group) showed a significantly higher concentration of KC/GRO, a potent neutrophil attractant, compared to the OVA/sal-sal control group.

## 4. Discussion

Nanomaterials are increasingly used for all kind of applications. When interaction with a biological system is expected, biocompatibility testing is essential. In the pharmaceutical and medical sector, nano-based drug delivery systems, in the form of nanoparticles carrying drugs, are suggested as alternative methods for targeted effective treatments with low doses. This type of drug delivery system has the advantage of improved drug stability and solubility, dose reduction, less frequent use, and less side effects [25,26]. The nanoparticles that are used for pulmonary drug delivery will come into contact with alveolar macrophages, which will identify them as foreign and try to clear them rapidly. Moreover, there are two biological barriers in the lungs which nano-based drug delivery systems need to cross: firstly, the mucus barrier, and secondly, the epithelial barrier of the airways [27]. Therefore, the size of the nanoparticles carrying the drugs is an important factor to pass these barriers. The behavior of inhaled particles in the lungs is very complex. It depends on several factors inherent to the particle, such as the particle size and solubility, but also on individual factors, such as air flow, breathing rate, and lung volume [28]. Yet, the consensus is that the size of the particles is the most critical factor for determining the distribution and the location of deposition in the lung. According to the convention of airborne particles, particles with a size of 100µm are generally deposited in the upper airways and the oropharyngeal region, and particles with a size of 10µm and smaller can reach the alveolar region [28,29,30]. Nanoparticles are defined as particles with a size of 1 to 100 nm and will definitely reach the alveolar region [31].

Asthma is widely spread in the human population; multiple phenotypes are described with different complex mechanisms of action [32]. One of the prominent features of asthma is airway narrowing due to persistent inflammation in the airway wall in the presence of inflammatory cells such as macrophages, mast cells, eosinophils, neutrophils, and lymphocytes [27,33,34]. The presence of a high number of eosinophils in the airways is a characteristic of OVA-induced asthma, and the rate of eosinophilia is correlated with the severity of disease [4,35]. It is well-known that eosinophils play a key role in inducing airway hyperresponsiveness (AHR) in atopic asthma [36,37,38]. Along with the cellular inflammation, selective T helper cell cytokines, such as interleukin (IL)-4, -5, -9, and -13, pro-inflammatory cytokines such as tumor necrosis factor-a (TNF-a) and IL-1b maintain the chronic inflammation [33,39]. To mimic the phenotype of IgE-mediated Th2-high asthma, ovalbumin (OVA) mouse models have been developed. Most often, aluminum hydroxide (an adjuvant) mixed with OVA is used to boost the intraperitoneal sensitization phase, after which an aerosol challenge with OVA results in an OVA-specific IgE response, with Th2 cells producing IL-4, IL-5, IL-10, and IL-13 and the typical pathologic and physiologic features of asthma [33]. In such models of OVA-induced asthma, several therapies have been investigated. Dorscheid et al. (2003) used an OVA model to investigate the role of corticosteroids to counteract airway epithelial damage and apoptosis [40]. They concluded that conventional therapies did not prevent the process of epithelial damage [40,41,42]. Kim et al. (2019) investigated whether inhalation exposure versus intranasal exposure to OVA resulted in differential outcomes [42]. They showed that pulmonary inflammation was significantly higher when inhalation exposure to OVA occurred. Recently, Wang et al. (2023) investigated a natural component, salidroside, which showed anti-asthmatic effects on OVA-induced asthmatic mice, with the results showing a significant therapeutic effect [43].

In most phenotypes of human asthma, short-acting β2 agonists are known to reverse the obstructive lung function during an asthma exacerbation [44]. In most cases, anti-asthmatic drugs are administered via inhalation, sometimes orally, or even intravenously [12]. The inhalation of drugs is preferred because it enables the drugs to directly reach the lungs. An example of a long-acting β2-agonist is formoterol. When formoterol is given in combination with budesonide, formoterol reduces the airway sensitivity, while budesonide reduces the inflammatory effect, but lowers the eosinophilic inflammation [45].

The drug delivered by specific systems should preferably target the diseased cells, while not affecting healthy cells, and preferably not show toxic effects while entering the systemic circulation [46]. A study on salbutamol encapsulation in liposomes showed effective distribution and a prolonged therapeutic effect [47]. Previously, we developed and optimized Lys-p(HEMA) NPs as a carrier for anti-asthmatic drugs, with biocompatible properties [20]. Bakan et al. (2019) also described the biodistribution of Lys-g-p(HEMA) NPs via the imaging of FITC-Lys-g- p(HEMA) by an in vivo imaging system (IVIS), and observed the NPs in different organs such as liver, kidney, heart, lung, and large intestine 6 h after administration [20]. For this study, we loaded the Lys-p(HEMA) NP with formoterol and investigated the effect on a mouse model of OVA-induced asthma. First, we tested the NPs loaded with formoterol for cytotoxic responses in different cell lines and can conclude that there no adverse effects occurred. As for any formulation, the amount of effective binding of the drug to the nanoparticle needs to be considered as well. We found that there was an initial burst release of the formoterol, but after a few hours, this stabilized. Even when the NPs with formoterol were stored for 10 days, the release was similar.

To test the effectiveness of our formulation, we have applied the NPs loaded with formoterol (ND) in an OVA asthma model and compared this to treatment with formoterol as it is normally administered (D). We used a preventive strategy, meaning that we administered the drugs 10 min before the specific aerosol OVA challenges. We also included multiple control groups of the non-loaded Lys-p(HEMA) NPs. From the data of the experimental model, we can conclude that the Lys-p(HEMA) NP (OVA/sal/-sal compared to OVA/NP-sal) did not cause any lung function alterations nor cellular inflammatory response itself, except for the production of KC/GRO in BAL. This is important because it is known that many factors can be affected by nanoparticles [12,15]. To our surprise, the formoterol both as a nanodrug (ND) and in a normal formulation (D) resulted in augmented asthmatic responses. The baseline lung function of the OVA/ND-OVA and OVA/D-OVA groups showed more tension in the lung structure, as shown by increased tissue damping (G) and tissue elasticity (H) compared to the positive control group (OVA/sal-OVA). Also, the lung volumes (FVC and FEV_0.1_) were significantly lower in the OVA-challenged groups that received the nanodrug and the normal drug formulation. Yet, the administration of the nanodrug or normal drug formulation in OVA-treated mice, did not result in altered airway hyperreactivity (both Rn as FEV_0.1_). Probably, the increased tissue tension and decreased lung volumes can be attributed to the significantly higher lung inflammation (macrophages and eosinophils) in the OVA-sensitized and -challenged groups, with the drug (ND or D) compared to the positive control group (OVA/sal-OVA). Clearly, the preventive therapeutic strategy that we applied resulted in an adverse health effect, rather than the expected improved health effects. By giving the bronchodilator formoterol in the ND or D formulation 10 min before the OVA aerosols challenges, we possibly “opened” the airways to a larger extent. This phenomenon needs to be investigated in more detail. An experimental set-up using a therapeutic design, hence administering the drugs after the OVA challenges, might clarify this.

Concerning the lung inflammatory response, mainly macrophages and eosinophils were increased in each OVA-challenged group. In the groups receiving the ND (OVA/ND-sal and OVA/ND-OVA), the neutrophils were also significantly increased compared to the control groups, which is confirmed by the higher concentration of KC/GRO in these (and other groups), indicating an irritative effect on the lungs by the ND and D in this experimental set-up. The eosinophilic inflammation, with the typical Th2 cytokine profile (IL-4, IL-5, and IL-13) is to be expected after OVA aerosol challenges, but the adverse increase in the cellular and biochemical response in the ND and D formulation with OVA treatment was similarly unexpected. Moreover, the unloaded nanoparticles (OVA/NP-OVA) also resulted in increased eosinophils. Abraha et al. (2004) already described a similar result as (S,S)-formoterol-increased IL-4 secretion in asthmatic mice [48]. Next to the eosinophils, the macrophages of the OVA-treated NP, ND, and D groups were also significantly increased compared to the positive control group OVA/sal-OVA, indicating a pro-inflammatory response.

In conclusion, the nanodrug carrier, Lys(Hema)-p, loaded with formoterol, does not show any signs of cytotoxicity in vitro. Yet, using a preventive strategy, in an in vivo mouse model of OVA-induced asthma with both the nanocarrier formulation and the normal drug, taking into account the particle characteristics, the route, and timing of administration, showed that increased innate and adaptive inflammation is induced, resulting in the worsening of lung function. This indicates that many challenges still need to be overcome before nano-based therapy can be applied in clinical practice.

## Figures and Tables

**Table 1:** Cytokine levels in BAL fluid.

Cytokines (pg/mL)	OVA/sal-sal	OVA/NP-sal	OVA/sal-OVA	OVA/NP-OVA	OVA/ND-sal	OVA/ND-OVA	OVA/D-sal	OVA/D-OVA
**IL-4**	<LOD	<LOD	2.52 ± 2.38	3.10 ± 2.38	<LOD	4.60 ± 1.86	<LOD	5.06 ± 1.21
**IL-5**	<LOD	<LOD	1.85 ± 1.03	2.62 ± 1.89	0.18 ± 0.16	6.74 ± 5.38	0.91 ± 1.35	14.57 ± 13.33 ^#^
**IL-13**	<LOD	<LOD	7.08 ± 5.67	10.04 ± 4.85	<LOD	10.26 ± 6.74	3.78 ± 1.60	23.49 ± 20.22
**IL-17A**	<LOD	<LOD	0.13 ± 0.09	0.31 ± 0.12	0.20 ± 0.19	0.51 ± 0.38	<LOD	0.31 ± 0.18
**IL-17F**	<LOD	<LOD	<LOD	<LOD	23.27 ± 15.58	<LOD	26.48 ± 24.94	<LOD
**IL-33**	10.07 ± 6.42	9.34 ± 10.78	34.62 ± 25.50	4.44 ± 2.23 ^##^	11.18 ± 8.36	11.68 ± 8.28	14.44 ± 6.19	11.76± 10.30
**IFN-γ**	<LOD	<LOD	<LOD	<LOD	<LOD	0.16 ± 0.15	<LOD	<LOD
**TNF-α**	2.16 ± 0.12	3.08 ± 1.23	3.99 ± 2.84	6.36 ± 5.44	13.21 ± 5.93 **	5.64 ± 3.36	1.75 ± 1.12	5.50 ± 1.60
**Il-6**	<LOD	<LOD	<LOD	<LOD	<LOD	5.22 ± 8.46	<LOD	<LOD
**KC/GRO**	14.52 ± 1.61	30.44 ± 12.59 *	51.20 ± 22.16 *	46.07 ± 18.84 *	52.80 ± 15.43 **	51.58 ± 19.48 *	12.39 ± 3.58	50.32 ± 21.92 *

Cytokine levels in bronchoalveolar lavage fluid were measured by using a U-plex assay (Meso Scale Diagnostics). D: drug; GRO: growth-regulated oncogene; IFN: interferon; IL: interleukin; KC: keratinocyte chemoattractant; LOD: limit of detection; ND: nanodrug formulation (Lys-p(HEMA) NP–formoterol); NP: nanoparticle; OVA: ovalbumin; sal: saline; TNF: tumor necrosis factor. The data are presented as mean ± SD (*n* = 5–6). * *p* < 0.05, ** *p* < 0.01 compared to OVA/sal-sal, and ^#^ *p* < 0.05, ^##^ *p* < 0.01 compared to OVA/sal-OVA. If the OVA/sal-sal negative control group or the OVA/sal-OVA positive control group was below the LOD, the statistical analysis could not be performed.

## Data Availability

The data presented in this study are available on request from the corresponding author.
